# Extending the Limits of Quantitative Proteome Profiling with Data-Independent Acquisition and Application to Acetaminophen-Treated Three-Dimensional Liver Microtissues* [Fn FN2]

**DOI:** 10.1074/mcp.M114.044305

**Published:** 2015-02-27

**Authors:** Roland Bruderer, Oliver M. Bernhardt, Tejas Gandhi, Saša M. Miladinović, Lin-Yang Cheng, Simon Messner, Tobias Ehrenberger, Vito Zanotelli, Yulia Butscheid, Claudia Escher, Olga Vitek, Oliver Rinner, Lukas Reiter

**Affiliations:** From the ‡Biognosys, Wagistrasse 25, CH-8952 Schlieren, Switzerland;; §Institute of Molecular Systems Biology, ETH Zurich, Auguste-Piccard-Hof 1, 8093 Zurich, Switzerland;; ¶Department of Statistics, Department of Computer Science, Purdue University, 150 N. University Street, West Lafayette, IN 47907-2068;; **InSphero AG, Wagistrasse 25, CH-8952 Schlieren, Switzerland

## Abstract

The data-independent acquisition (DIA) approach has recently been introduced as a novel mass spectrometric method that promises to combine the high content aspect of shotgun proteomics with the reproducibility and precision of selected reaction monitoring. Here, we evaluate, whether SWATH-MS type DIA effectively translates into a better protein profiling as compared with the established shotgun proteomics.

We implemented a novel DIA method on the widely used Orbitrap platform and used retention-time-normalized (iRT) spectral libraries for targeted data extraction using Spectronaut. We call this combination hyper reaction monitoring (HRM). Using a controlled sample set, we show that HRM outperformed shotgun proteomics both in the number of consistently identified peptides across multiple measurements and quantification of differentially abundant proteins. The reproducibility of HRM in peptide detection was above 98%, resulting in quasi complete data sets compared with 49% of shotgun proteomics.

Utilizing HRM, we profiled acetaminophen (APAP)^1^-treated three-dimensional human liver microtissues. An early onset of relevant proteome changes was revealed at subtoxic doses of APAP. Further, we detected and quantified for the first time human NAPQI-protein adducts that might be relevant for the toxicity of APAP. The adducts were identified on four mitochondrial oxidative stress related proteins (GATM, PARK7, PRDX6, and VDAC2) and two other proteins (ANXA2 and FTCD).

Our findings imply that DIA should be the preferred method for quantitative protein profiling.

Quantitative mass spectrometry is a powerful and widely used approach to identify differentially abundant proteins, *e.g.* for proteome profiling and biomarker discovery (1). Several tens of thousands of peptides and thousands of proteins can be routinely identified from a single sample injection in shotgun proteomics (2). Shotgun proteomics, however, is limited by low analytical reproducibility. This is due to the complexity of the samples that results in under sampling (supplemental Fig. 1) and to the fact that the acquisition of MS2 spectra is often triggered outside of the elution peak apex. As a result, only 17% of the detectable peptides are typically fragmented, and less than 60% of those are identified. This translates in reliable identification of only 10% of the detectable peptides (3). The overlap of peptide identification across technical replicates is typically 35–60% (4), which results in inconsistent peptide quantification. Alternatively to shotgun proteomics, selected reaction monitoring (SRM) enables quantification of up to 200–300 peptides at very high reproducibility, accuracy, and precision (5–8).

Data-independent acquisition (DIA), a novel acquisition type, overcomes the semistochastic nature of shotgun proteomics (9–18). Spectra are acquired according to a predefined schema instead of dependent on the data. Targeted analysis of DIA data was introduced with SWATH-MS (19). For the originally published SWATH-MS, the mass spectrometer cycles through 32 predefined, contiguous, 25 Thomson wide precursor windows, and records high-resolution fragment ion spectra (19). This results in a comprehensive measurement of all detectable precursors of the selected mass range. The main novelty of SWATH-MS was in the analysis of the collected DIA data. Predefined fragment ions are extracted using precompiled spectrum libraries, which results in SRM-like data. Such targeted analyses are now enabled by several publicly available computational tools, in particular Spectronaut^2^, Skyline (20), and OpenSWATH (21). The accuracy of peptide identification is evaluated based on the mProphet method (22).

We introduce a novel SWATH-MS-type DIA workflow termed hyper reaction monitoring (HRM) (reviewed in (23)) implemented on a Thermo Scientific Q Exactive platform. It consists of comprehensive DIA acquisition and targeted data analysis with retention-time-normalized spectral libraries (24). Its high accuracy of peptide identification and quantification is due to three aspects. First, we developed a novel, improved DIA method. Second, we reimplemented the mProphet (22) approach in the software Spectronaut (www.spectronaut.org). Third, we developed large, optimized, and retention-time-normalized (iRT) spectral libraries.

We compared HRM and state-of-the-art shotgun proteomics in terms of ability to discover differentially abundant proteins. For this purpose, we used a “profiling standard sample set” with 12 non-human proteins spiked at known absolute concentrations into a stable human cell line protein extract. This resulted in quasi complete data sets for HRM and the detection of a larger number of differentially abundant proteins as compared with shotgun proteomics. We utilized HRM to identify changes in the proteome in primary three-dimensional human liver microtissues after APAP exposure (25–27). These primary hepatocytes exhibit active drug metabolism. With a starting material of only 12,000 cells per sample, the abundance of 2,830 proteins was quantified over an APAP concentration range. Six novel NAPQI-cysteine proteins adducts that might be relevant for the toxicity of APAP were found and quantified mainly on mitochondrion-related proteins.

## EXPERIMENTAL PROCEDURES

### 

#### 

##### Materials

Conalbumin was purchased from Fluka. Ribonuclease B, beta casein, fibrinogen, and myoglobin were purchased from SIGMA AltrichSt. Louis, MO. 6 Bovine Tryptic Digest Equal Molar Mix was purchased from Bruker, Billerica, MA. The HEK-293cells were kindly provided by Dr. Audrey van Drogen (ETH, Zurich). Iodoacetamide, tris(2-carboxyethyl)phosphine, trifluoroacetic acid, acetonitrile (ACN), HPLC water, ammonium bicarbonate, acetaminophen, and urea were purchased from SIGMA-Aldrich. Trypsin sequencing grade was purchased from Promega, Madison, WI. RapiGest was purchased from Waters, Milford, MA. Synthetic, heavy-labeled peptides were purchased from Thermo Scientific, Waltham, MA.

##### Sample Preparation

A 15-cm dish of confluent HEK-293 cells was lysed by resuspension in 8 m urea and 0.1 m ammonium bicarbonate (to 1 μg/μl protein). The lysate was reduced with 5 mm tris(2-carboxyethyl)phosphine for 1 h at 37 °C. Subsequently, the lysate was alkylated with 25 mm iodoacetamide for 20 min at 21 °C. The lysate was diluted to 2 m urea and digested with trypsin at a ratio 1:100 (enzyme to protein) at 37 °C for 15 h. The samples were spun at 20,000 g at 4 °C for 10 min. The peptides were desalted using C18 MacroSpin columns from The Nest Group, Southborough, MA according to manufacturer's instructions. After drying, the peptides were resuspended in 1% ACN and 0.1% formic acid. 100 μg of conalbumin and ribonuclease B, beta-casein, fibrinogen, and myoglobin were prepared as described for the cell lysate above. The Biognosys' HRM Calibration Kit was added to all of the samples according to manufacturer's instructions (required for the DIA analysis using Biognosys' Spectronaut) Schlierern, ZH, Switzerland.

3D InSight™ Human Liver Microtissues (InSphero AG InSphero, Schlieren, ZH, Switzerland) consisting of primary human hepatocytes (lot IZT) and primary human non-parenchymal cells (lot JJB) were cultivated in GravityTRAP™ plates with 70 μl 3D InSight™ Human Liver Maintenance Medium (InSphero AG) per well (27). The liver microtissues were treated at cultivation day 5 with 0, 1.5, 4.5, 13.7, 41.2, 123, 5, 370.4, 1,111.1, 3,333.3, and 10,000 μm APAP (dissolved in the medium) for 3 days without redosing. Biological triplicates of each concentration were measured with CellTiter-Glo® ATP-assay (Promega). 12 single microtissues from each condition were pooled in an Eppendorf tube. Next, the cells were spun at 200g for 5 min at room temperature and then washed twice with PBS with spinning as before. The cells were lysed in 20 μl of 10 m urea, 0.1 m ammonium bicarbonate, and 0.1% RapiGest, sonicated for 3 min and centrifuged at 16,000 g for 2 min at 18 °C. Subsequently, the samples were prepared as described for the cell lysate above.

##### Mass Spectrometric Acquisition

1 μg of the samples was analyzed on a self-made analytical column (75 μm × 30 cm) packed with 3 μm Magic C18AQ medium (Bruker) at 50 °C, using an Easy-nLC connected to a Q Exactive mass spectrometer (Thermo Scientific). The peptides were separated by a 2 h linear gradient of from 5 to 35% ACN with 0.1% formic acid at 300 nl/min, followed by a linear increase to 98% ACN in 2 min and 98% for 8 min. For DDA acquisition, the “fast” method from Kelstrup was used with the following alterations (28). The full scan was performed between 400–1,220 *m/z*. The automatic gain control target for the MS/MS scan was set to 5e5. Stepped collision energy was 10% at 25%. The HRM DIA method consisted of a survey scan at 35,000 resolution from 400 to 1,220 *m/z* (automatic gain control target of 5*10^6^ or 120ms injection time). Then, 19 DIA windows were acquired at 35,000 resolution (automatic gain control target 3e6 and auto for injection time) (supplemental Table 1). Stepped collision energy was 10% at 25%. The spectra were recorded in profile type. The MS/MS spectra were recorded from 200 to 1800 *m/z.* SRM was performed on a linear gradient form 5 to 35% ACN of 10 min on a Thermo Scientific TSQ Vantage using a 10-cm column as described above, unscheduled with 40 ms dwell time and 0.8 Thompson isolation width. 0.5 μm sample was spiked with the stable isotope standards. The raw mass spectrometric data were stored at the public repository PeptideAtlas (http://www.peptideatlas.org, No. PASS00589, the username is PASS00589 and the password is WF6554orn).

##### Mass Spectrometric Raw Data Analysis

The DIA data were analyzed with Spectronaut 5, a mass spectrometer vendor-independent software from Biognosys. The default settings were used for the Spectronaut search. Retention time prediction type was set to dynamic iRT (correction factor for window 1). Decoy generation was set to scrambled (no decoy limit). Interference correction on MS2 level was enabled. The false discovery rate (FDR) was set to 1% at peptide level. The DDA spectra were analyzed with the MaxQuant Version 1.4.1.2 analysis software using default settings with the following alterations (29). The minimal peptide length was set to 6. Search criteria included carbamidomethylation of cysteine as a fixed modification, oxidation of methionine and acetyl (protein N terminus) as variable modifications. The mass tolerance for the precursor was 4.5 ppm and for the fragment ions was 20 ppm. The DDA files were searched against the human UniProt fasta database (state 29.04.2013, 20,254 entries), the spike in proteins (12 entries), and the Biognosys iRT peptide sequences (11 entries). The identifications were filtered to satisfy FDR of 1% on peptide and protein level.

##### Spectral Library Generation

For generation of the spectral libraries, 24 DDA measurements of the “profiling standard sample set” or six DDA measurements of the microtissues were performed. DDA spectra were analyzed as described above, and a spectral library was generated using spectral library generation in Spectronaut, similar to SpectraST (30). Apart from annotation of precursors and fragment ions the library also contained normalized retention times (iRT). For the generation of the assays of the APAP adducts, a variable modification (C_8_H_7_NO_2_) was generated modifying the cysteine. The carbamidomethyl cysteine was set to variable modification.

##### Data Filtering and Processing

For the profiling standard sample set, peptides shared between HEK-293 proteome, the spike-in proteins, and contaminants from MaxQuant were removed. For precursors that were identified multiple times within one MS run using MaxQuant, the most intense identification was selected. Full peptide profiles were defined as full peptide precursor profiles not merging modified species and charge states. The microtissue candidate lists were generated with FDR of 0.05 (adj. *p* value) and fold change to control of more than 50%. Gene ontology enrichment was performed using DAVID Bioinformatics Resources 6.7 (31).

##### Linear Mixed Effect Model Tests for Differential Abundance

For both DDA and DIA, the log_2_-intensities of the peptides were summarized across all the samples and runs, and separately for each protein, in a linear mixed model implemented in MSstats (32). The model is summarized below. Here [GRAPHIC] denotes sample [GRAPHIC], [GRAPHIC] denotes precursor [GRAPHIC], and [GRAPHIC] denotes run [GRAPHIC] denotes the log-intensities of the peptides, and [GRAPHIC] denotes the random nonsystematic deviations of the observed intensities that are not explained by the systematic sources of variation.

Model: Linear Mixed Effects Model Implemented in MSstats

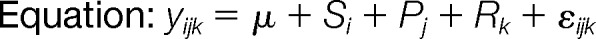


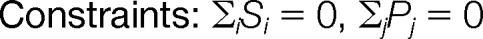


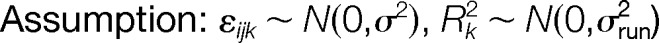


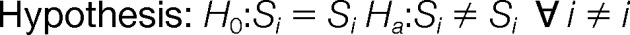


Model Fit. Restricted Maximum Likelihood Estimator (RMLE)

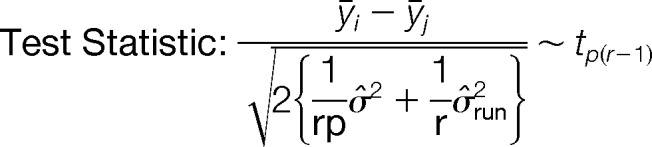


Notation: *r* = *the number of runs* = *3, p* = *the numbers of precursors, σ̂^2^* = *the RMLE of the variance of the random error, σ̂_run_^2^* = *the RMLE of the variance of Run*

Note: The test statistic is given for the special case of a balanced dataset with no missing values.

The model was used to perform all the 28 possible pairwise comparisons of samples, separately for each protein. The p values were adjusted for multiple testing using the Benjamini-Hochberg method (33). For unfeasible comparisons caused by missing values, the p value was set to 1, reflecting inability to detect differential abundance. The R package pROC was used to obtain the receiving operator characteristic curves, and calculate the areas under the curve. The areas under the curves obtained with two acquisition strategies were compared using the DeLong's test (34).

## RESULTS

### 

#### 

##### Development of a Novel DIA Method

**Fig. 1.**
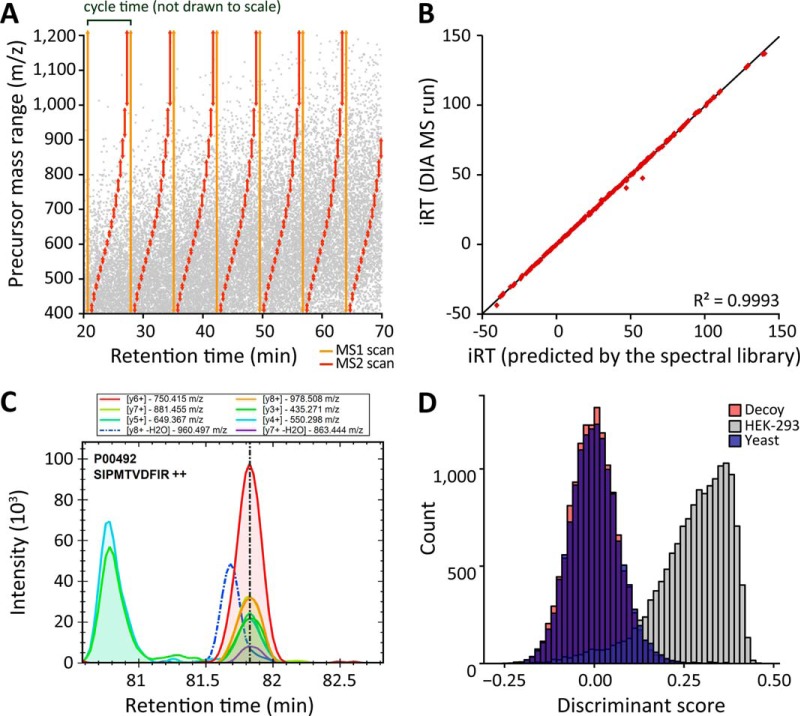
**Novel DIA method for HRM and analysis using Spectronaut.** (*A*) The novel SWATH-MS type DIA method consists of one survey scan and 19 DIA windows with adapted swath size to the precursor density implemented on a Thermo Scientific Q Exactive mass spectrometer. (*B*) iRT used in postacquisition prediction of peptide elution time in an HRM run. Using a static extraction window of 20min, 200 sampled, identified peptides were visualized. (*C*) SRM-like, direct visualization of extracted ion currents. Example of a fragment ion with an interfering signal (dotted line) as automatically detected by Spectronaut shown. Vertical line indicates iRT value of peptide assay (*D*). The histogram shows the normalized discriminant score used for the FDR (Spectronaut's Cscore) of targets, decoys and truly absent peptides (sample 3 profiling standard sample set). The use of scrambled peptide sequences as decoys (red) statistically mimics truly absent yeast peptides (blue). This is verified using a spectral library for yeast peptides in the analysis of acquisitions derived from the human cell line (gray). Note the clear separation of decoys and targets.

A novel acquisition method was developed to enable high performance DIA on a Q Exactive instrument. It consists of one survey scan (MS1), and 19 variable DIA swaths (MS2) that adapt to the different total ion current complexity of precursor-ion mass range (Fig. 1A and supplemental Table 1). This method is utilizing the new DIA workflow of Xcalibur 3.0, which enables direct measurement of large DIA windows with automatic gain control. This reduces the cycle time (3.5 s) by a factor of 1.7 as compared with the previously used MS1/AIF (All Ion Fragmentation) method for DIA^3^, while matching the cycle time of the original SWATH-MS acquisition method (19). The DIA method is optimized to the used LC peak capacity resulting on average in six to eight data points per peak.

##### HRM Data Analysis Workflow

To extract intensities of peptides from spectra of the DIA data, we have developed a mass spectrometer vendor-independent software called Spectronaut. Peak picking and scoring is largely similar to SRM (22), with notable exceptions. First, fragment ions can be defined post acquisition. Second, the large-scale nature of DIA experiments facilitates the use of robust and efficient machine learning algorithms for peak picking and scoring. Retention time information can be used to accurately predict the elution of the peptides by in-measurement calibration of iRT values. This can be thought of as a computational scheduling procedure, which allows the usage of ion current extraction windows with the width of a few percent of the gradient expected to satisfy over 99% of peptides (Fig. 1*B*). Third, peptide quantification is further improved by means of an automated, advanced interference detection algorithm (Fig. 1*C*). The method of decoy generation is crucial for the accuracy of the resulting FDR (35). The model implemented in Spectronaut was validated by searching yeast peptides in DIA-spectra derived from a human cell line. The score distribution of the decoys was compared with the score distributions of the spectra of the truly absent (yeast) peptides (Fig. 1*D*).

##### Profiling Standard Sample Set, a Proteome Profiling Simulation

**Fig. 2.**
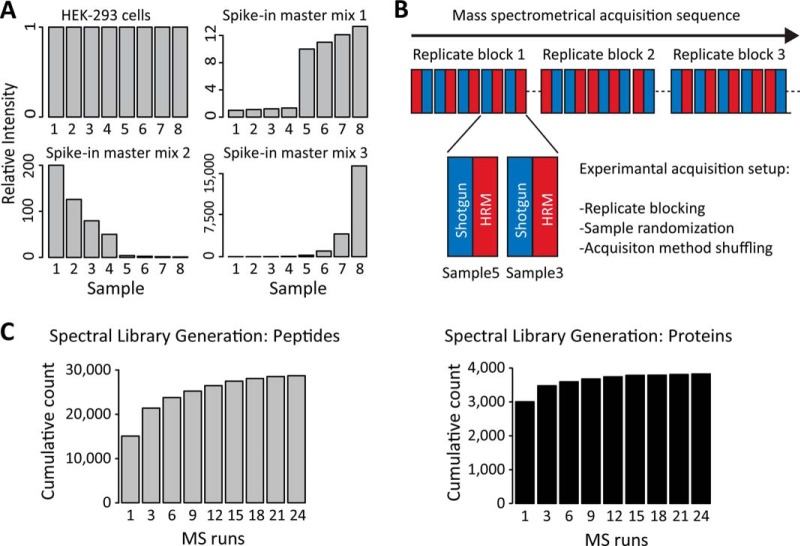
**Design of the HRM and shotgun proteomics comparison experiment.** (*A*) The profiling standard sample set consisted of eight samples with a constant background (HEK-293 cells) and 12 non-human proteins spiked in three master mix groups. The lowest concentration was set to the value 1. (*B*) Spectral acquisition of the profiling standard sample set was performed on a Q Exactive mass spectrometer in shotgun proteomics and HRM mode in a block-randomized manner. (*C*) The spectral library for the targeted search was generated from shotgun proteomics MS runs of the profiling standard sample set. The peptide and protein counts of the spectral libraries with increasing shotgun proteomics MS runs are shown in the bar plots.

To benchmark the profiling performance of HRM and to compare with the established shotgun proteomics, we generated a series of controlled mixtures termed the profiling standard sample set. 12 non-human proteins (supplemental Table 2) were spiked into a constant background (HEK-293). The profiling standard sample set presented two main challenges for mass spectrometric profiling: detection of a small number of differentially abundant proteins among a background of not-changing proteins and precise relative quantification. The 12 spike-in proteins were grouped into three master mixes. Two master mixes were diluted to introduce small differences in concentrations (10 and 60% changes) at limit of detection and at 10- and 50-fold higher concentrations. The third master mix was pipetted in a fourfold dilution series starting at the limit of detection (Fig. 2*A*). Spectra from the profiling standard sample set were acquired on a Q Exactive mass spectrometer in DDA and DIA mode in three technical replicates. The order of MS runs was block-randomized to avoid bias (Fig. 2*B* and supplemental Table 3) (36).

##### Comprehensive Spectral Library for the Profiling Standard Sample Set

For the targeted analysis of the HRM spectra, we generated a comprehensive spectral library using shotgun proteomics consisting of 29,246 peptide assays (23,290 proteotypic) for 3,836 protein groups with an average of 28% sequence coverage (supplementary file HRM-Spectral-libraries.xlsx).

Since the proposed HRM approach can only identify and quantify peptides and proteins for which the assays are present in the spectral library, the content of the spectral library strongly influences the final result. In order to assess the importance of acquiring redundant shotgun proteomics runs at the library generation step, we generated a series of spectral libraries from an increasing number of DDA runs. The FDR was controlled at protein level (37). The cumulative number of proteins included in the library started to plateau after 12 to 15 runs, the peptides after 21 to 24 runs. Therefore, additional runs of shotgun proteomics increased the peptide coverage per protein in the library (Fig. 2*C*). Before the inclusion into the library, the peptide identifications were filtered for the presence of at least six suitable fragment ions and for restrictions on variance in iRT retention shift (five standard deviations).

##### Profiling Data Analysis and Normalization of the Profiling Standard Sample Set

The raw DIA spectra were analyzed in targeted mode with Spectronaut using the spectral library described above. No feature alignment was used. The dynamic retention time extraction window for the targeted HRM search was about 3% gradient, achieved by high-resolution iRT. The combined scores implemented in Spectronaut resulted in a clear separation of the target and decoy distributions (supplemental Fig. 2). On average, 28,610 peptides were identified per measurement in HRM. The raw DDA spectra of the profiling standard sample set were analyzed with the software MaxQuant (29). No feature alignment was used. On average 17,547 peptides were identified per measurement in shotgun proteomics. Overall, the HRM approach identified on average 60% more peptides in a single run than shotgun proteomics. This is primarily due to the comprehensive nature of precursor fragmentation with DIA and to the comprehensive spectral library. For the two data sets, local normalization was performed on the peptide quantities to compensate for loading differences and spray bias (38) (supplemental Fig. 3, stored in Profiling-Standard-Data-Set-tables.xlsx).

##### Quasi Complete Data Set of HRM Profiling and Quantitative Precision of the Profiling Standard Sample Set

**Fig. 3.**
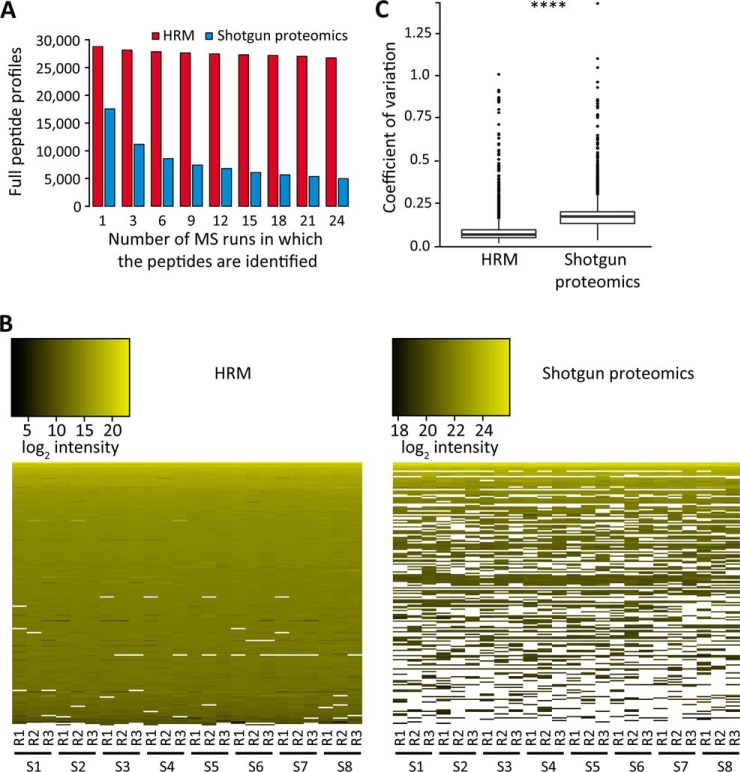
**Comparison of the quantitative measurements generated by HRM and shotgun proteomics for the background (HEK-293).** (*A*) Number of peptide precursors that were identified in all the indicated MS runs without missing values. (*B*) Coefficients of variation of 4,360 peptides from the background, which were identified in both shotgun proteomics and HRM, and quantified in all the 24 runs. The box plots show significant difference for HRM and shotgun proteomics (**** *t* test, *p* value < .0001). (*C*) 200 peptides were randomly selected from all the peptides that were identified by both HRM and shotgun proteomics. The selected peptides were ordered in a heat map vertically by intensity and horizontally by sample. White spots indicate missing intensities among the selected peptides.

Reproducibility of identification was analyzed for HRM and shotgun proteomics. Remarkably, the HRM data set contained over five times more full profiles of peptides of 24 MS runs (HRM contained 26,738 full profiles of peptides of 3,690 proteins and shotgun proteomics 4,974 full profiles of 1,522 proteins) (Fig. 3*A*). HRM generated a quasi complete data set, with only 1.6% missing values (10% of those are spike-in proteins) compared with 51% missing values for shotgun proteomics (Fig. 3*B*). This demonstrates the excellent ability of the targeted HRM workflow to identify peptides repeatedly and completely.

**Fig. 4.**
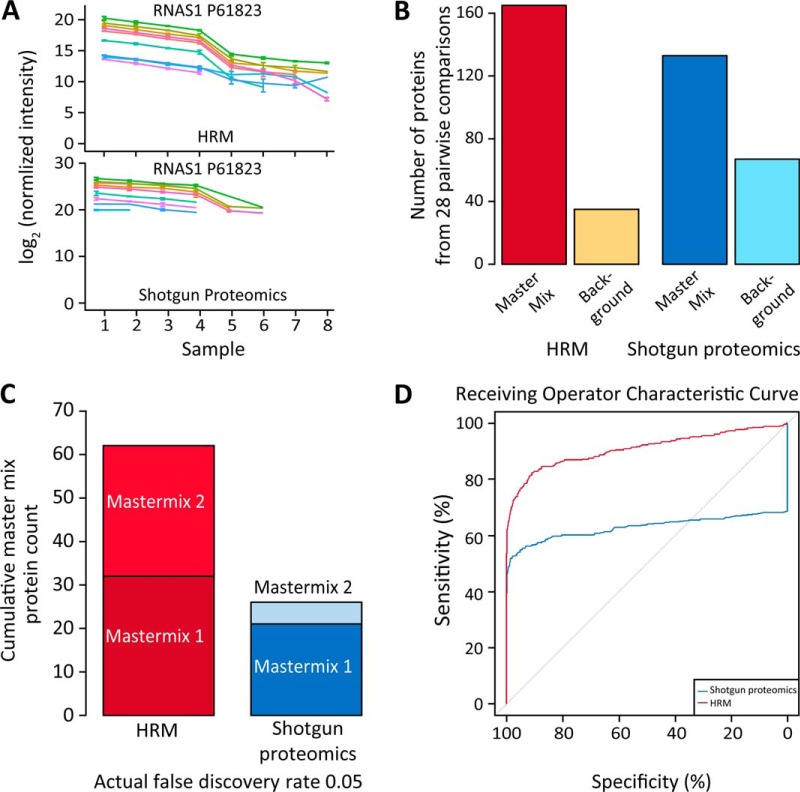
**Model-based detection of differentially abundant proteins in the 28 possible pairwise comparisons of the profiling standard sample set.** (*A*) Profile of the peptides of the spike in protein RNAS1 P61823 of master mix 2. The error bars show the standard deviation. (*B*) The top 200 candidate proteins ranked by adjusted *p* value. (*C*) The cumulative detection of spike in proteins of the 28 pairwise comparisons after setting the cutoff of the actual FDR to 0.05 (the number of false positives divided by the number of candidates, from the ground truth). (*D*) Receiver operating characteristic (ROC) curve of detecting differentially abundant proteins, obtained with respect to the known changes in protein concentrations. Each point on the curve corresponds to a different FDR cutoff, and relates the sensitivity (equivalently, true positive rate TPR) and specificity (equivalently, true negative rate TNR). The ROC curve uses all the proteins in the profiling standard sample set” (*p* value ≤ 2.22 10^-16) (34).

To compare the quantitative precision of shotgun proteomics and HRM, we calculated the coefficients of variation (CV) for all the background peptides that were quantified in both experiments and detected in all the runs. The CVs of the HRM profiles were 53% lower than of the same profiles of shotgun proteomics (average CV of 8.45% for HRM, 17.8% for shotgun proteomics) (Fig. 3*C*). The results for shotgun proteomics are consistent with the previously published results, which reported around 10–20% CV for consecutive technical measurement replicates on the same mass spectrometer (4). Additionally, the CVs for HRM were found to be lower than those for shotgun proteomics over the whole intensity range (supplemental Fig. 4). Detailed analysis of the master mix protein profiles clearly showed that HRM created fewer missing values and more homogenous peptide profiles, especially for master mixes 2 and 3 (Fig. 4*A* and supplemental Fig. 5).

##### Statistical Analysis of the Profiling Standard Sample Set

We analyzed quantitative profiles of the identified peptides to evaluate the ability of the two methods to detect differentially abundant proteins in the profiling standard sample set. The comparisons were performed separately for shotgun proteomics and for HRM, using a family of linear mixed effects models implemented in MSstats (32). For each protein and each of the 28 possible pairs of comparisons among the eight mixtures, the model was used to estimate the log fold change (Profiling-Standard-Data-Set-tables.xlsx).

The result of model-based comparisons of all pairs of mixtures were used to generate candidate lists of 200 proteins, ranked by the adjusted *p* values in all the 28 pairwise comparisons, separately for HRM and shotgun proteomics. The cutoff of 200 proteins was selected, primarily for reasons of practical interpretability. For each protein and each pairwise comparison, the quality of the conclusions of differential abundance was determined based on the ground truth, *i.e.* based on the known amount of changes in the spiked master mix and the background proteins. (Fig. 4*B*). Shotgun proteomics identified nearly double the number of background proteins.

The comparisons at the actual FDR of 0.05 showed that the HRM approach identified more than twice as many spike in proteins (Fig. 4*C*). The receiving operator characteristic (ROC) revealed the stronger overall performance of HRM (Fig. 4D and supplemental Table 4). Neither HRM nor shotgun proteomics was able to detect concentration changes of 10–30% with the three replicates. HRM had a better ability to detect concentrations changes of 60% and higher (supplemental Fig. 6).

##### Treatment of Three-Dimensional Human Liver Microtissue with Acetaminophen

**Fig. 5.**
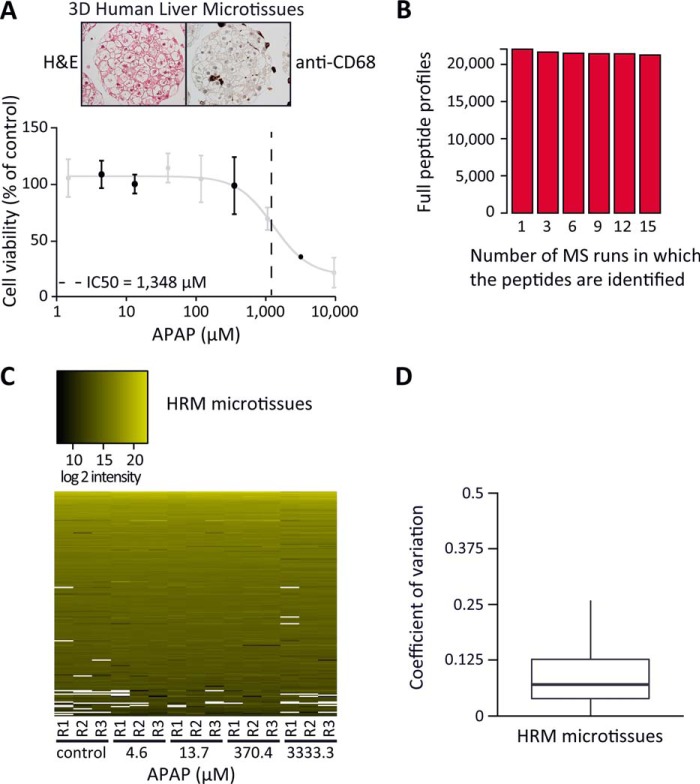
**HRM profiling of APAP treated three-dimensional human liver microtissues.** (*A*) Hematoxylin and eosin (H&E) stained three-dimensional microtissue shows liver-like morphology. CD68 positive Kupffer cells are present and distributed throughout the microtissue. Below, the viability of the microtissues after 72 h APAP treatment was analyzed using a luminescent cell viability assay. Three subtoxic concentrations (4.6, 13.7, 370.4 μm) and one toxic APAP concentration (3,333.3 μm) were analyzed by HRM (black) (12,000 cells per sample). (*B*) Number of peptides identified in all the indicated MS runs without missing value. (*C*) 200 peptides were randomly selected from all the peptides that were identified by HRM. The selected peptides were ordered in a heat map vertically by intensity and horizontally by sample. (*D*) CVs of peptides identified and quantified by HRM are visualized in a box plot. The CVs were calculated from the subset of peptides identified in the technical triplicates of the samples without missing values.

The model system of three-dimensional human liver microtissues from InSphero was used to identify relevant proteomic changes upon physiological APAP treatment using HRM (Fig. 5*A*, upper images). In order to test the effects of APAP on the proteome, the liver microtissues (12,000 cells per sample) were treated with a concentration range of APAP and the corresponding control. The viability was assessed by an intracellular ATP-assay post treatment (Fig. 5*A*, lower graph). A clear dose-dependent effect of APAP on the viability was observed, with a half maximal inhibitory concentration of 1,348 ìM APAP (95% confidence interval 870 to 2,089 μm). Subsequently, three subtoxic (4.6, 13.7, and 370.4 μm) and one toxic (3,333.3 μm) APAP concentration were chosen for HRM profiling.

##### Profiling of Acetaminophen-Treated Three-Dimensional Human Liver Microtissue

A spectral library was generated from the microtissue samples containing 23,108 peptides (17,290 proteotypic) of 2,830 protein groups with on average 28% sequence coverage (supplementary file HRM-Spectral-libraries.xlsx). The microtissue samples were acquired in DIA mode using a block-randomization (supplemental Table 5). The spectra were analyzed with Spectronaut. On average 22,454 peptides were identified per measurement. The high reproducibility of peptide detection resulted in a quasi complete data set, despite the presence of biological and technical variation. 1.3% of values were missing from 15 measurements (Figs. 5*B* and 5*C*). The CVs of peptides identified in three technical replicates were on average 11.2% (Fig. 5*D*).

**Fig. 6.**
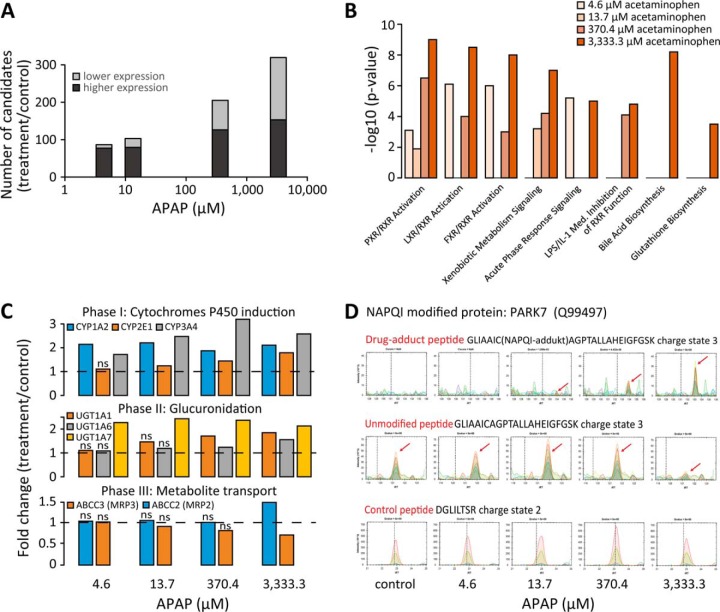
**Biostatistical analysis of three-dimensional liver microtissues after APAP treatment.** (*A*) The candidate's lists of the treatments compared with control. (*B*) Selected pathways, significantly enriched in the candidate lists of the APAP treatment. The *p* values indicate statistically significant overrepresentation of the genes in a given process. (*C*) Induction of the biotransformation phase I, II and III enzymes upon APAP exposure. The dashed line indicates unchanged expression compared with control. The “n.s.” marks not significantly detected changes. (*D*) NAPQI modification of PARK7 was detected at the cysteine 106. A reduction of the unmodified peptide was observed. HRM XICs shown of the NAPQI-modified, unmodified and a control peptide. The arrows indicate the peptide signals.

The treatment series was analyzed using the linear mixed effects models of MSstats. Increasing concentrations of APAP resulted in increasing numbers of regulated proteins compared with control (Fig. 6*A*). Besides known proteins and pathways (reviewed in (39)), novel ones and secondary effects as bile acid biosynthesis and glutathione biosynthesis were detected. (Fig. 6*B*, supplementary file Micro-tissue-Data-Set-tables.xlsx). APAP exposure induced elevated levels of eight phase I cytochromes P450 (CYP). CYP2E1 was induced at the highest toxic concentration, as described (40) (Fig. 6*C*, upper panel). Additionally, glutathione S-transferases were induced, which are responsible of APAP-conjugation to glutathione (39). Phase II metabolizing proteins for APAP-glucuronidation were identified at significantly elevated levels post APAP exposure. UGT1–7 was significantly up-regulated starting from the lowest APAP concentration, whereas UGT1–1 and UGT1–6 were up at higher concentrations (Fig. 6*C*, middle panel). The phase III metabolite transporter for biliary secretion (MRP2) was significantly up-regulated, and the transporter for basolateral excretion (MRP3) was significantly down (Fig. 6*C*, lower panel). The differential expression of the candidates apolipoprotein B-100 and heat shock 70 kDa protein 1A/1B was confirmed using stable isotope standard SRM (supplemental Fig. 7). At toxic APAP concentrations, cell-death-associated proteins, such as cytochrome *c* (41) and JNK-associated protein SPAG9 were significantly up-regulated.

##### Acetaminophen Adduct Quantification on Mitochondrial Proteins

Phase I enzymes partly metabolize APAP into its reactive NAPQI intermediate, which is conjugated to Glutathione and to cellular proteins. In the DDA measurements of the microtissue treatment, NAPQI modification of cysteines was identified at the highest APAP concentration on four mitochondria related proteins (GATM, PARK7, PRDX6, and VDAC2) and additionally on ANXA2 and FTCD (supplementary file Micro-tissue-Data-Set-tables.xlsx). Interestingly, HRM analysis of the modification specific peptides revealed, that NAPQI-adducts are clearly detectable at physiological relevant, nontoxic concentrations of 13.7 μm APAP. The modified peptides eluted 9.8 min (±0.9 min for 95% confidence interval) after the unmodified form (Fig. 6*D* and supplemental Fig. 8).

## DISCUSSION

We reported a direct comparison of quantification performance of HRM and shotgun proteomics using a profiling standard sample set. The HRM workflow outperformed shotgun proteomics in number of consistently identified peptides, in precision of quantification, and in detection of differentially abundant proteins.

The most obvious and direct advantage of the HRM approach for multisample profiling is the quasi complete data matrix that can be obtained without any interrun alignment. Missing data is the greatest limitation of shotgun proteomics for quantification (42). The HRM approach essentially solves this limitation. The MS data can be further processed using interrun alignment methods, *e.g.* as described for HRM^4^ or for shotgun proteomics (43, 44). We believe that the ability of HRM to side-step the need for alignment leads to better and more replicable data sets.

A major difference between HRM and shotgun proteomics is the set of the quantified ions. In shotgun proteomics, the quantification is performed on the precursor (MS1) level, in HRM on the level of the fragments ions (MS2). Quantification on the fragment ion level is less susceptible to interferences, as presence of simultaneous interferences for all fragment ions is unlikely. A typical shotgun proteomics method uses ∼25% of the acquisition time for MS1 spectra that are used for quantification. The DIA method introduced here uses ∼95% of the acquisition time for MS2 spectra that are used for quantification. The comparison of CVs suggests that quantification on the MS2 level is substantially more precise (Fig. 3*C*). The findings are consistent with Egertson *et al.*, who compared the intensity of MS1 and of MS2 (MSX measurement) signals (18). The extracted ion currents of candidates can be directly visualized and manually validated like in SRM, *i.e.* a peptide from P2666 could be quantified down to 100 attomol on column (supplemental Fig. 9).

Using HRM profiling with APAP-treated microtissues, proteins from all three phases of drug metabolism were identified as regulated. Clearly, the HRM technology allows unbiased protein profiling for discovery experiments, revealing novel mode-of-actions or -toxicity upon drug treatment.

It is believed that the main mechanism of cell death induced by APAP is the binding of the reactive NAPQI intermediate to cellular proteins, potentially affecting their function (39). However, despite more than 40 years of research, so far no NAPQI-adduct sites on human proteins were mapped. We have profiled NAPQI adduct sites on six different proteins. PARK7 plays an important role in mitochondrial oxidative stress (45); the drug-adduct detected at PARK7's active site (C106) likely inhibits its function (46). PRDX6 is involved in the protection of cells against mitochondrial dysfunction in liver injury (47). The voltage-dependent anion channel (VDAC2) was shown to be involved in response to oxidative stress by mediating translocation of GSK3B into the mitochondrion (48). Annexin A2 has been implicated in diverse cellular processes like in oxidative stress and apoptosis (49). These findings strongly suggest that relevant targets of APAP protein modification were identified, which might directly contribute to APAP-mediated cell death.

The reproducibility and high throughput of HRM have the potential to reposition mass spectrometry-based proteomics in the biological sciences. In our proposed workflow, shotgun proteomics does not become obsolete but is used for the purpose where it has its real strength: the generation of exhaustive spectral libraries that serve as templates for HRM targeted data analysis. The application of DIA acquisition for high content discovery is a novel approach with the potential for further performance increase that has not been fully exploited yet.

## Supplementary Material

Supplemental Data

## Footnotes

^2^ Bernhardt, O. M., Selevsek, N., Gillet, L. C., Rinner, O., Picotti, P., Aebersold, R., and Reiter, L. (2012) Spectronaut A fast and efficient algorithm for MRM-like processing of data independent acquisition (SWATH-MS) data. Proceedings of the 60th ASMS Conference on Mass Spectrometry and Allied Topics, 2012, Vancouver, BC, Canada.

^3^ Bruderer, R. M., Miladinović, S. M., Bernhardt, O. B., Rinner, O., Aebersold, R., and Reiter L. (2013) Highly multiplexed protein profiling across large sets of samples: A comparison of SWATH acquisition with shotgun LC-MS/MS. 61th ASMS Conference on Mass Spectrometry and Allied Topics, 2013, Minneapolis Convention Center, Minneapolis, MN.

^4^ Bernhardt, O. B., Bruderer, R. M., Gandhi, T. P., Miladinović, S. M., Ossola, R., Butscheid, Y., Rinner, O., and Reiter, L. (2013) A novel algorithm for protein profiling based on cross-run correlations in SWATH data implemented in Spectronaut. 61th ASMS Conference on Mass Spectrometry and Allied Topics, June 9–13, 2013, Minneapolis, MN.
