# Supplementary material for: Extending the Limits of Quantitative Proteome Profiling with Data-Independent Acquisition and Application to Acetaminophen-Treated Three-Dimensional Liver Microtissues
Source: Mol Cell Proteomics. 2015 Feb 27;14(5):1400–10. doi: 10.1074/mcp.M114.044305 (PMC4424408; doi:10.1074/mcp.M114.044305)
Supplement: Supplemental Data [file supp_14_5_1400__index.html]

Extending the limits of quantitative proteome profiling with data-independent acquisition and application to acetaminophen treated 3D liver microtissues — Extending the Limits of Quantitative Proteome Profiling with Data-Independent Acquisition and Application to Acetaminophen-Treated Three-Dimensional Liver Microtissues — HRM Proteome Profiling — Supplemental Data 

# Extending the Limits of Quantitative Proteome Profiling with Data-Independent Acquisition and Application to Acetaminophen-Treated Three-Dimensional Liver Microtissues

## Supplemental Data

**Files in this Data Supplement:**

- Bruderer-Supplemental-Information - File contains the supplemental figures and legends.
- HRM-Spectral-libraries.xlsx - File contains the Spectral libraries for the HRM analyses.
- Profiling-Standard-Data-Set-tables.xlsx - File contains the analyses of the Profiling Standard Sample Set of the HRM and Shotgun proteomics.
- Micro-tissue-Data-Set-tables.xlsx - File contains the analyses of the Microtissue experiment of the HRM and Shotgun proteomics.
