# Supplementary material for: Extending the Limits of Quantitative Proteome Profiling with Data-Independent Acquisition and Application to Acetaminophen-Treated Three-Dimensional Liver Microtissues
Source: Mol Cell Proteomics. 2015 Feb 27;14(5):1400–10. doi: 10.1074/mcp.M114.044305 (PMC4424408; doi:10.1074/mcp.M114.044305)
Supplement: Supplemental Data [file supp_M114.044305_mcp.M114.044305-1.pdf]

Supplementary Tables and Figures

Suppl. Figure 1. The occupancy of the TOP12 method during spectral acquisition of the HEK-293 digest in a 2 hour gradient. For most of the acquisition time, the mass spectrometer was reaching its maximal sequencing capacity, and therefore was under sampling the digest of HEK-293 cells (bar plot from RawMeat 2.1, Vast Scientific).

Suppl. Figure 2. Target-decoy separation. Histogram of the normalized discriminant score (Spectronaut's Cscore) of the target peptides (grey) and the decoys generated (red) from the whole "Profiling Standard Sample Set", as obtained by the Spectronaut software. Note the clear separation of the distributions of scores of targets and decoys. This reflects the high quality of the spectral library and the DIA method.

Suppl. Figure 3. Normalization of MS quantities. After the initial feature detection and quantifications, HRM and shotgun proteomics were normalized to correct for small differences in loading as well as spray fluctuations occurring in label free proteomics. The log intensities of all detected peptide precursor intensities are shown.

Suppl. Figure 4. Visualization of the coefficients of variation in the "Profiling Standard Sample Set", among precursors identified and quantified in both shotgun proteomics and HRM mode in all the 24 runs. The precursors were binned into 10 ranges of mean intensity percentiles. The error bars show the standard errors of the mean. The intensity range spans almost four orders of magnitude.

Suppl. Figure 5. Profiles of spike in proteins of the three master mixes. The average and standard deviation of the normalized intensities per sample were plotted for the indicated proteins in HRM and shotgun proteomics. Note, that the HRM approach had better coverage of the low abundant identification.

Suppl. Figure 6. Detection of differentially abundant proteins in the "Profiling Standard Sample Set", with focus on the true fold change. (A) Detection of 10-30 % changes of master mix1 in samples 5 - 8. The identified master mix 1 proteins were plotted against the actual FDR. (B) Detection of 60 % changes of concentration in samples 1 - 4. (C) Detection of ≥4 fold change in all the 28 pairwise comparisons.

Suppl. Figure 7. Extracted ion chromatograms (XICs) of liver microtissue NAPQI-adduct targets. The XICs of NAPQI-adduct peptides, its unmodified counterpart and a control peptide are visualized by Spectronaut. The arrows indicate peptides signals of the NAPQI-modified and unmodified peptide. The X-axis shows normalized retention time iRT and the Y-axis shows ion intensity.

Suppl. Figure 8. SRM analysis of the microtissue candidates Apolipoprotein B-100 and Heat shock 70 kDa protein 1A/1B. SRM measurements were performed in triplicate using microtissue samples at 0 µM and 3,333.3 µM APAP treatment. For Apolipoprotein B-100 (FSVPAGIVIPSFQALTAR charge state 2) and Heat shock 70 kDa protein 1A/1B (LLQDFFNGR charge state 2), isotopically labelled reference peptides (R+10 Da) were used. The average ratio is shown and for SRM standard deviation of the triplicate.

Suppl. Figure 9. XICs of peptides of the "Profiling Standard Sample Set". (A) The XIC from Biognosys' Spectronaut show the XICs of the endogenous protein VCP (P55072) (involved in neurodegenerative

diseases) with peptide LGDVISIQPCPDVK in charge state 2. VCP is a stable protein identified in the background (HEK-293). (B) XIC for master mix 1 protein Beta-Lactoglobulin (P02754) peptide VLVLDTDYK charge state 2 (sample 1-8 replicate 1). (C) XIC of master mix 2 protein Fibrogen G (P12799) peptide LDGSLDFK charge state 2 (sample 1-8 replicate 1). (D) XIC of master mix 3 protein beta Casein (P02666) peptide GPFPIIV charge state 2 (sample 1-8 replicate 1), additionally, a zoom of the XIC is shown. The lowest signal in sample 1 corresponds to 100 attomol. The X-axis shows normalized retention time iRT and the Y-axis shows ion intensity.

Supplementary Table 1: Novel DIA method. DIA swaths for all ion fragmentation on the Q Exactive that were used for spectral acquisition of the "Profiling Standard Sample Set". The range and window size is shown in m/z.

Window Nr.	Range	Window size
1	400-430	30
2	428-459	31
3	457-483	26
4	481-506	25
5	504-531	27
6	529-554	25
7	552-576	24
8	574-600	26
9	598-624	26
10	622-650	28
11	648-676	28
12	674-704	30
13	702-735	33
14	733-771	38
15	769-810	41
16	808-856	48
17	854-914	60
18	912-1000	88
19	998-1220	222

Supplementary Table 2: Overview of the sample series constituting the “Profiling Standard Sample Set”. Samples 1-8 consisted of a stable background (HEK-293 cell line), and were spiked with 12 non-human proteins in three master mixes. The concentrations are given in relative and absolute concentrations.

	**Master mix1**<br>P02754<br>P80025<br>P00921<br>P00366<br>P02662		**Master mix2**<br>P61823<br>P02789<br>P12799<br>P02676<br>P02672		**Master mix3**<br>P02666<br>P68082		**Background**<br>HEK-293	
**Sample**	**relative**	**fmol/ul**	**relative**	**fmol/ul**	**relative**	**fmol/ul**	**relative**	**µg/ul**
1	1	1.5	200	100	1	0.05	1	0.5
2	1.1	1.65	125.99	62.995	4	0.2	1	0.5
3	1.21	1.815	79.37	39.685	16	0.8	1	0.5
4	1.33	1.995	50	25	64	3.2	1	0.5
5	10	15	4	2	256	12.8	1	0.5
6	11.01	16.515	2.52	1.26	1024	51.2	1	0.5
7	12.11	18.165	1.59	0.795	4096	204.8	1	0.5
8	13.33	19.995	1	0.5	16384	819.2	1	0.5

Supplementary Table 3: Acquisition sequence order of mass spectrometric MS run in the “Profiling Standard Sample Set”. Samples S1-8 were measured in triplicates with HRM and shotgun proteomics (8x3x2 = 48 MS runs). The order of the runs was block-randomized, such that HRM and shotgun proteomics acquisitions of each sample were next to each other.

**Acquisition sequence**	**Sample**	**Replicate**	**Method**
1	2	1	HRM
2	2	1	shotgun proteomics
3	6	1	shotgun proteomics
4	6	1	HRM
5	1	1	shotgun proteomics
6	1	1	HRM
7	4	1	shotgun proteomics

8	4	1	HRM
9	5	1	shotgun proteomics
10	5	1	HRM
11	8	1	HRM
12	8	1	shotgun proteomics
13	7	1	shotgun proteomics
14	7	1	HRM
15	3	1	shotgun proteomics
16	3	1	HRM
17	4	2	HRM
18	4	2	shotgun proteomics
19	8	2	shotgun proteomics
20	8	2	HRM
21	6	2	HRM
22	6	2	shotgun proteomics
23	1	2	shotgun proteomics
24	1	2	HRM
25	3	2	shotgun proteomics
26	3	2	HRM
27	7	2	shotgun proteomics
28	7	2	HRM
29	5	2	HRM
30	5	2	shotgun proteomics
31	2	2	shotgun proteomics
32	2	2	HRM
33	1	3	HRM
34	1	3	shotgun proteomics
35	3	3	shotgun proteomics
36	3	3	HRM
37	7	3	shotgun proteomics
38	7	3	HRM
39	2	3	shotgun proteomics
40	2	3	HRM
41	4	3	shotgun proteomics
42	4	3	HRM
43	5	3	HRM
44	5	3	shotgun proteomics
45	6	3	shotgun proteomics
46	6	3	HRM
47	8	3	shotgun proteomics
48	8	3	HRM

Supplementary Table 4: Table of the area under the curve (AUC) of detecting differentially abundant proteins, obtained with respect to the known changes in protein concentrations of the "Profiling Standard Sample Set" and its confidence interval.

Method	Shotgun proteomics	HRM
AUC	62.80 %	90.80 %
Confidence Interval of AUC	(57.9-67.7 %)	(88.6-93.1 %)

Supplementary Table 5: The order of spectral acquisition in the human liver microtissue toxicology study. The samples were block-randomized, and profiled in three technical replicates.

**Acquisition sequence**	**Sample**	**Replicate**	**Method**	**APAP (uM)**
1	S4	1	HRM	13.7
2	S7	1	HRM	370.4
3	S9	1	HRM	3,333.3
4	S3	1	HRM	4.5
5	S1	1	HRM	0
6	S3	2	HRM	4.5
7	S1	2	HRM	0
8	S7	2	HRM	370.4
9	S4	2	HRM	13.7
10	S9	2	HRM	3,333.3
11	S1	3	HRM	0
12	S3	3	HRM	4.5
13	S9	3	HRM	3,333.3
14	S4	3	HRM	13.7
15	S7	3	HRM	370.4

HRM proteome profiling

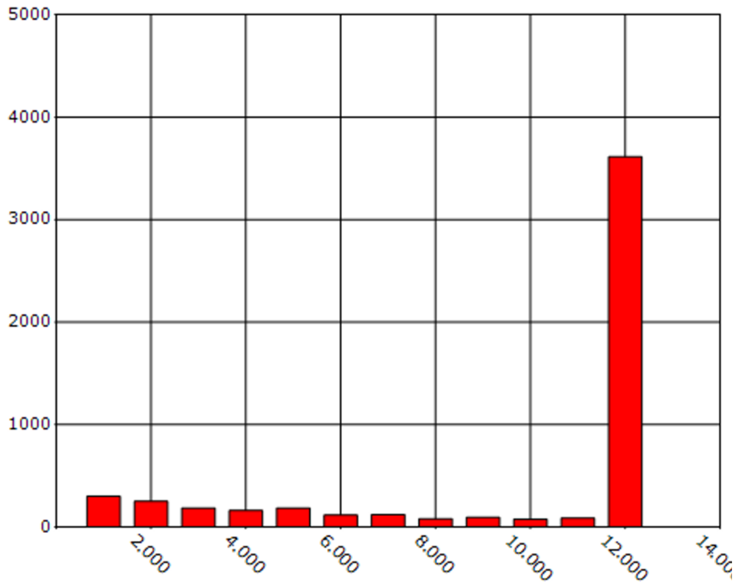

Suppl. Figure 1

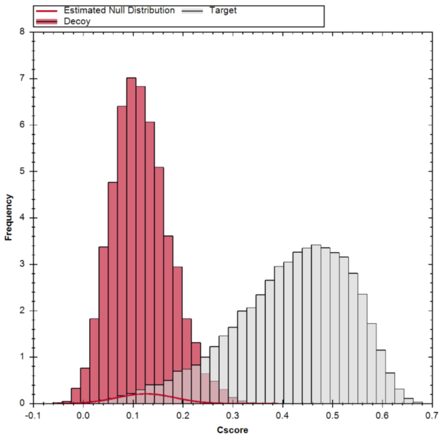

Suppl. Figure 2

HRM proteome profiling

Shotgun proteomics

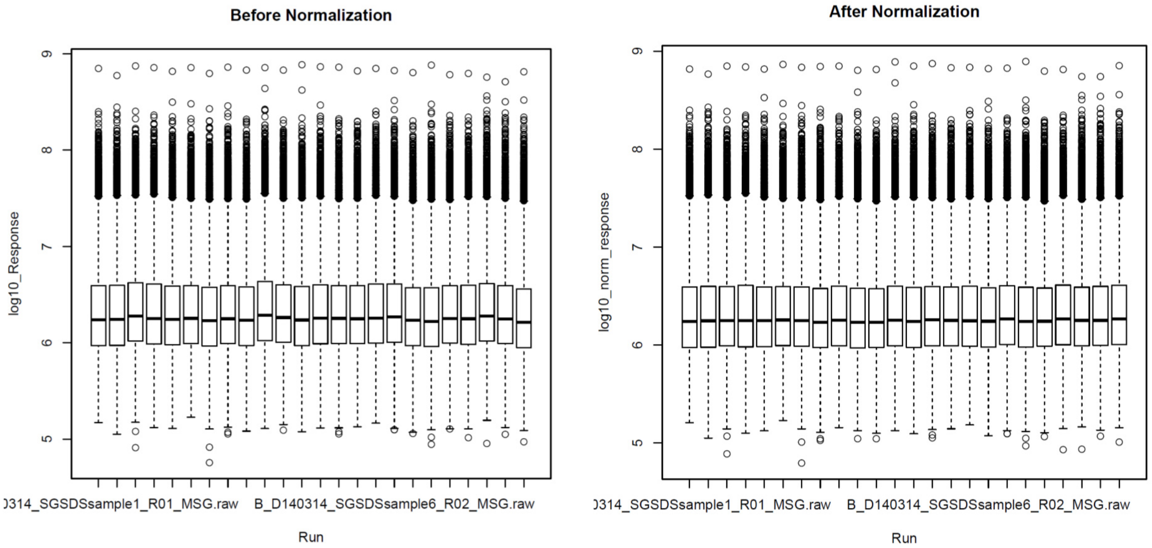

HRM

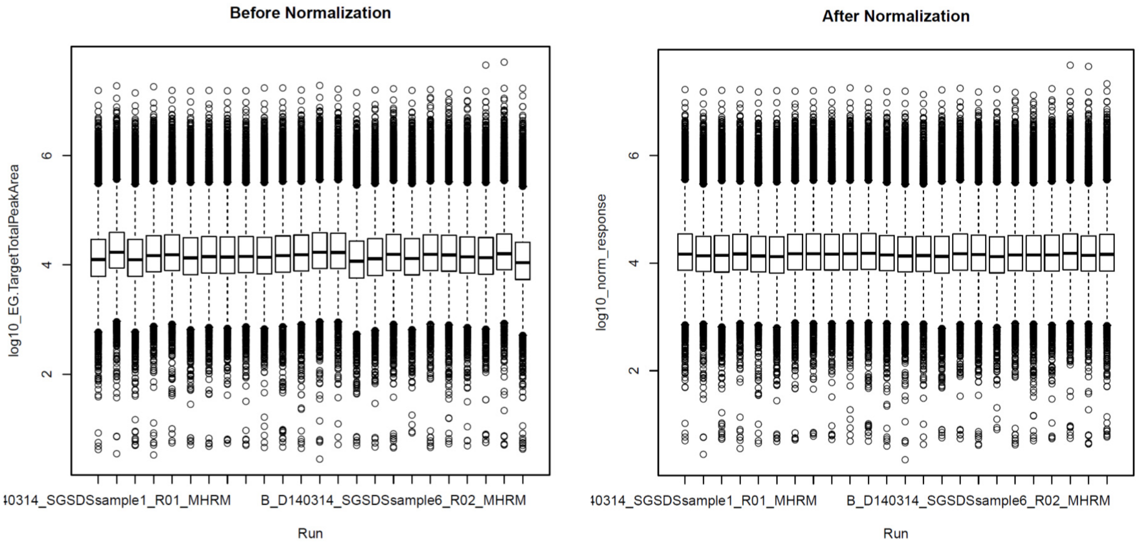

Suppl. Figure 3

HRM proteome profiling

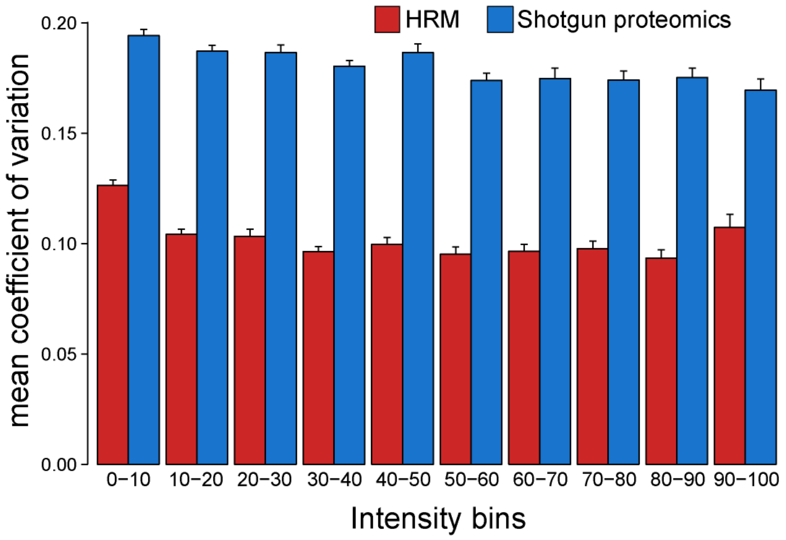

Suppl. Figure 4

HRM proteome profiling

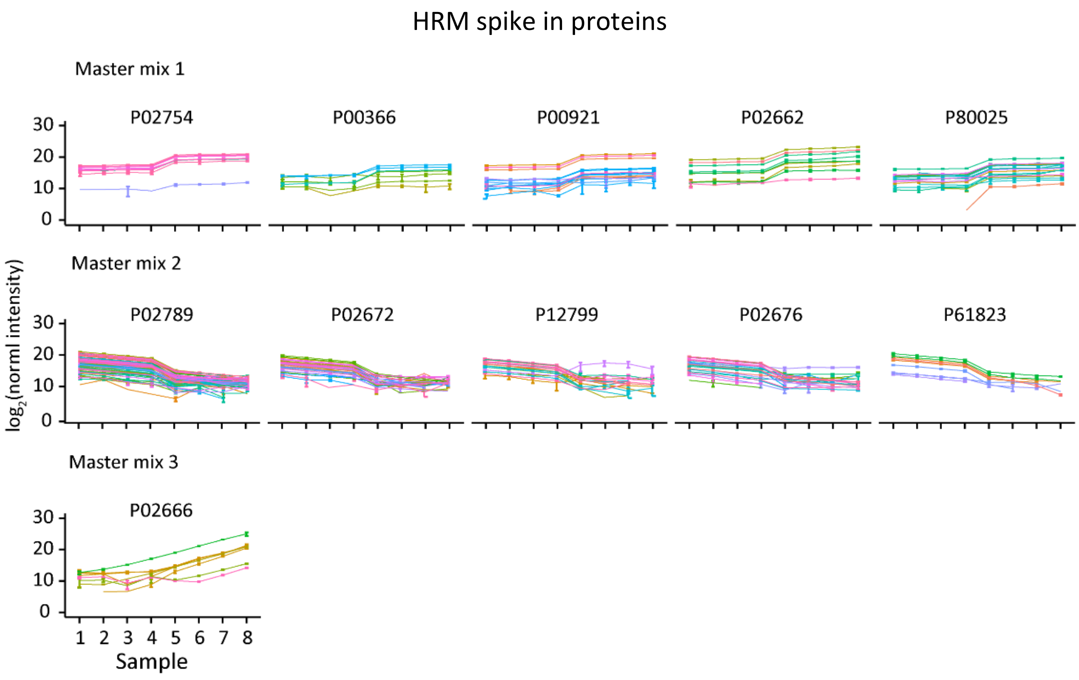

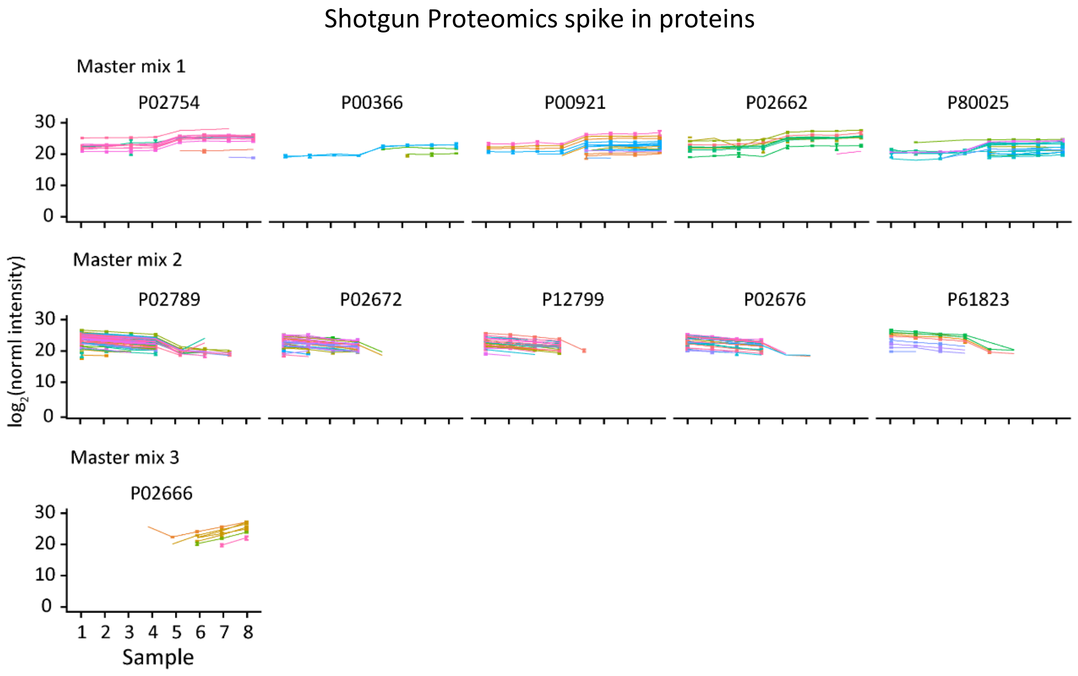

Suppl. Figure 5

HRM proteome profiling

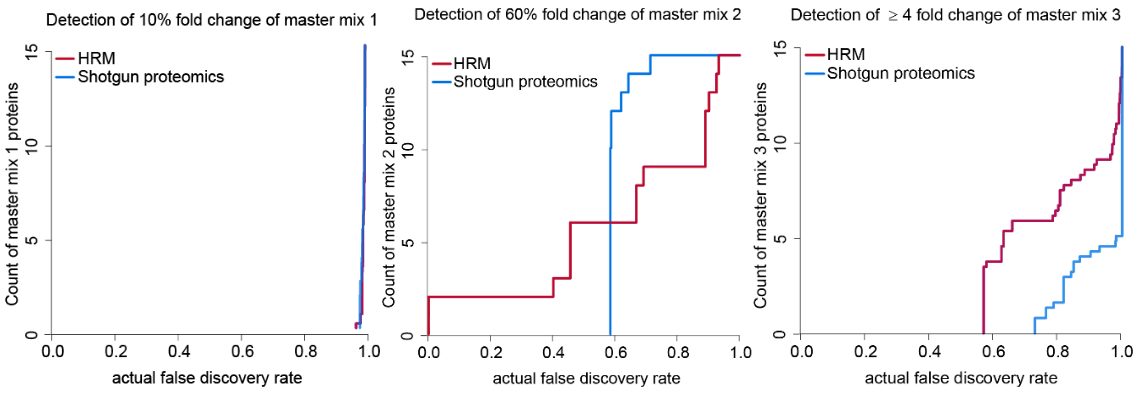

Suppl. Figure 6

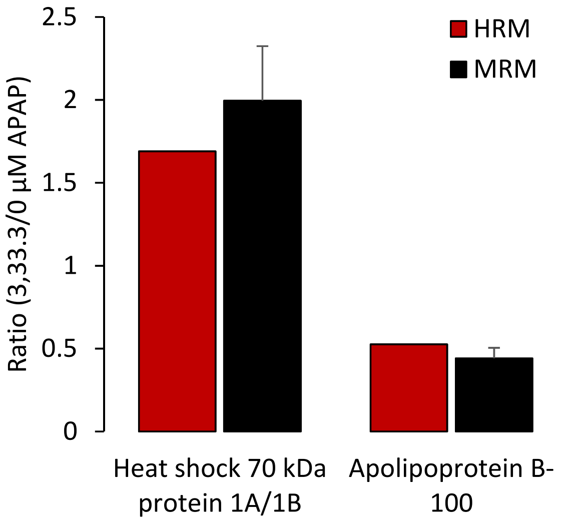

Suppl. Figure 7

HRM proteome profiling

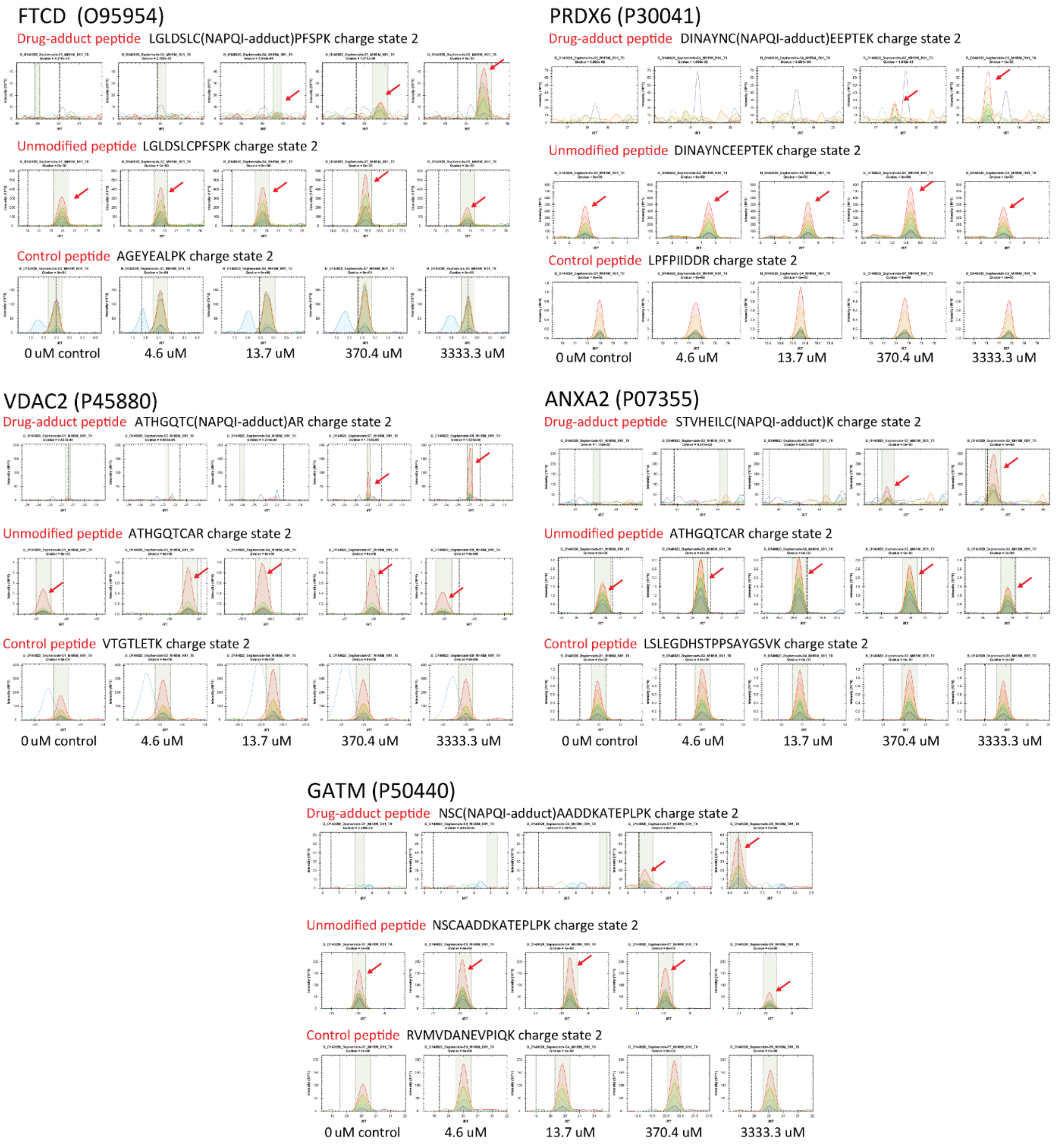

Suppl. Figure 8

a VCP (P55072) peptide LGDVISIQPCPDVK in charge state 2 (background HEK-293) Sample 1 to Sample 8 from top left with all three replicates.

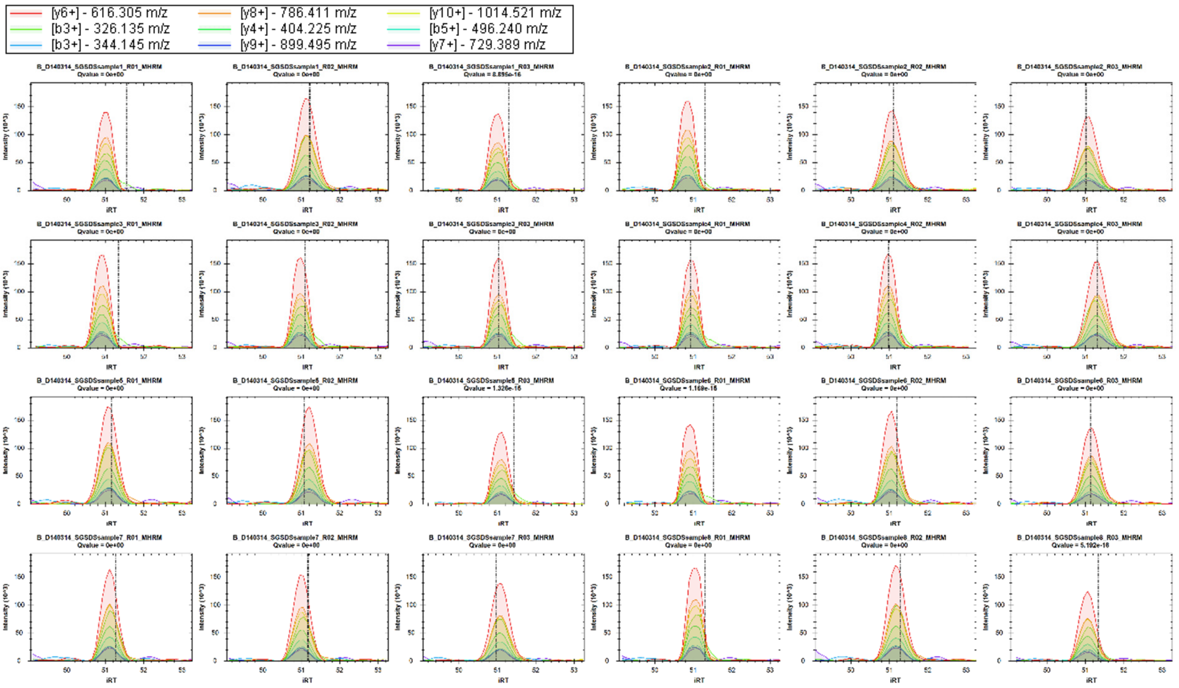

b B-Lactoglobulin (P02754) peptide VLVLDTDYK charge state 2 (master mix 1) sample 1 to sample 8 (replicate 1).

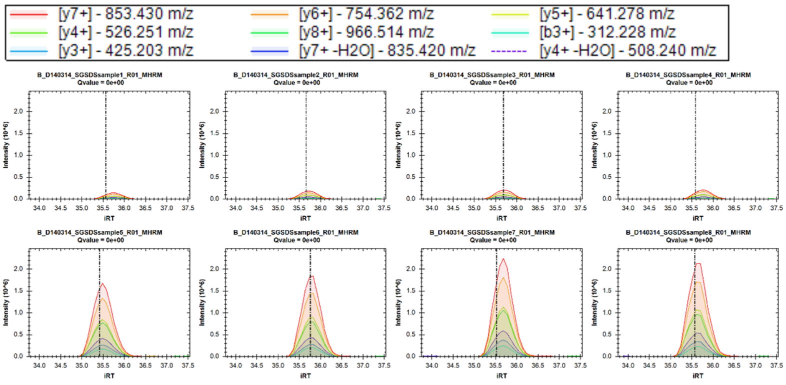

Suppl. Figure 9 a b

c Fibrogen G (P12799) peptide LDGSLDFK charge state 2 (master mix 2) sample 1 to sample 8 (replicate 1).

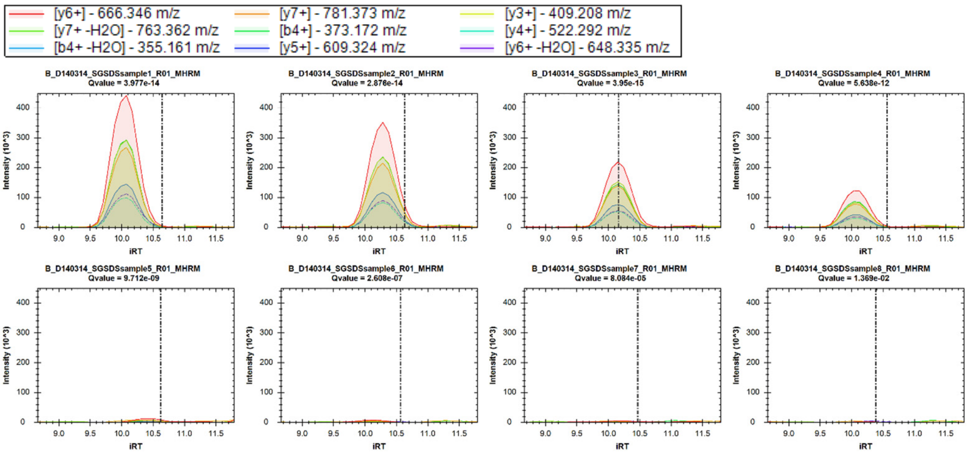

d β-Casein (P02666) peptide GPFPIIV charge state 2 (master mix 3) sample 1 to sample 8 (replicate 1).

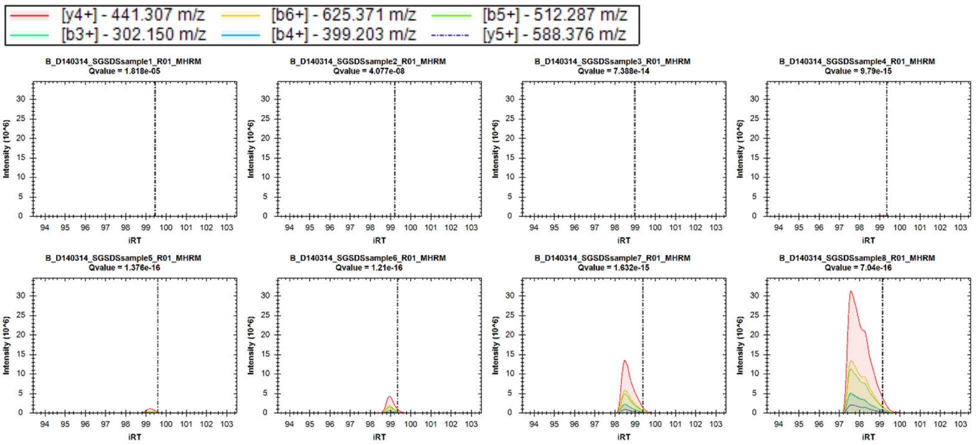

Zoom to visualize low intensity signals in sample 1 to sample 4

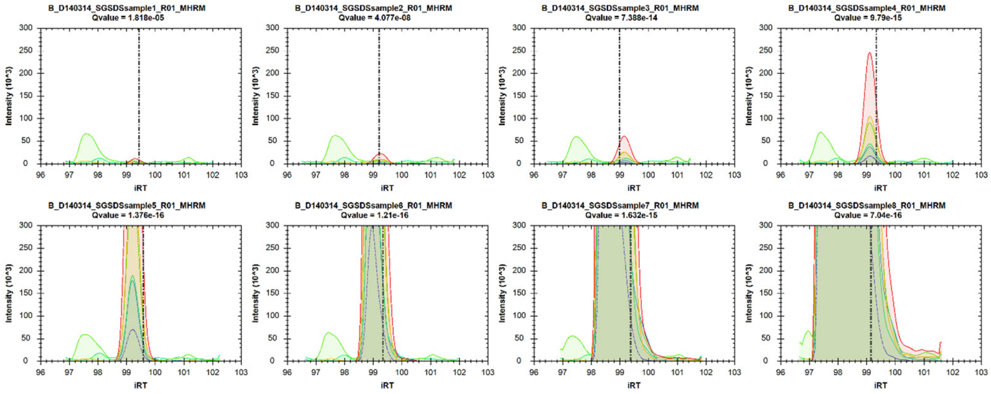

Suppl. Figure 9 c d
